# Targeting GLI Transcription Factors in Cancer

**DOI:** 10.3390/molecules23051003

**Published:** 2018-04-24

**Authors:** Miroslava Didiasova, Liliana Schaefer, Malgorzata Wygrecka

**Affiliations:** 1 Department of Biochemistry, Faculty of Medicine, Universities of Giessen and Marburg Lung Center, Friedrichstrasse 24, 35392 Giessen, Germany; malgorzata.wygrecka@innere.med.uni-giessen.de; 2 Institute of Pharmacology and Toxicology, Goethe University, Theodor Stern Kai 7, 60590 Frankfurt am Main, Germany; schaefer@med.uni-frankfurt.de; 3 The German Center for Lung Research, 35392 Giessen, Germany

**Keywords:** cancer, glioma-associated oncogene homolog, hedgehog signaling, GLI inhibitors, cancer stem cells

## Abstract

Aberrant activation of hedgehog (Hh) signaling has been observed in a wide variety of tumors and accounts for more than 25% of human cancer deaths. Inhibitors targeting the Hh signal transducer Smoothened (SMO) are widely used and display a good initial efficacy in patients suffering from basal cell carcinoma (BCC); however, a large number of patients relapse. Though SMO mutations may explain acquired therapy resistance, a growing body of evidence suggests that the non-canonical, SMO-independent activation of the Hh pathway in BCC patients can also account for this adverse effect. In this review, we highlight the importance of glioma-associated oncogene (GLI) transcription factors (the main downstream effectors of the canonical and the non-canonical Hh cascade) and their putative role in the regulation of multiple oncogenic signaling pathways. Moreover, we discuss the contribution of the Hh signaling to malignant transformation and propose GLIs as central hubs in tumor signaling networks and thus attractive molecular targets in anti-cancer therapies.

## 1. Hedgehog Signaling in Cancer

**Figure 1:**
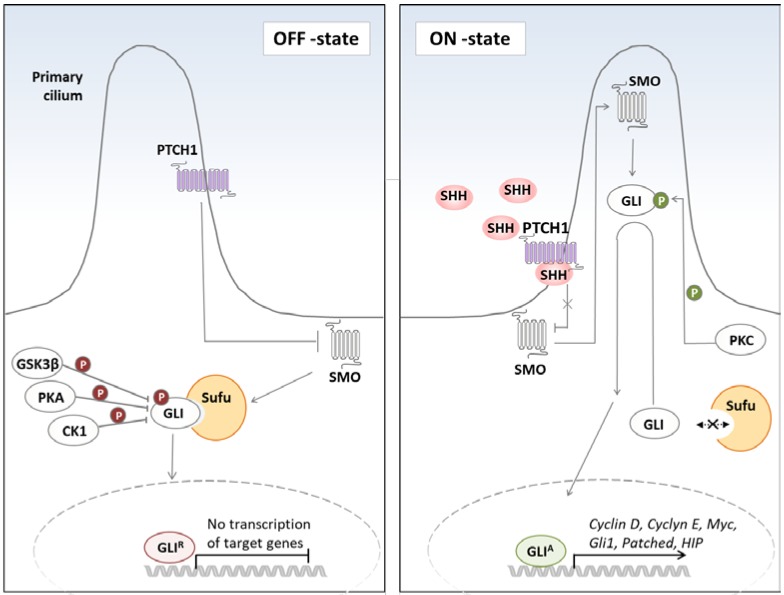
Mechanism of Hedgehog pathway activation. In the absence of the Hh ligand (**left** panel), PTCH1, which is found in the primary cilium, binds to SMO and prevents its transclocation into the cilium. This leads to the sequestration of GLIs in the cytoplasm, their association with the negative regulator SUFU, phosphorylation by GSK3β/PKA/CK1 kinases, and subsequent cleavage into repressor forms (GLI^R^). In the presence of the Hh ligand (**right** panel), SMO inhibition by PTCH1 is relieved, and SMO translocates to the primary cilium and prevents GLI2 and GLI3 cleavage. GLI proteins dissociate from SUFU, are phosphorylated by PKC, and converted into their active forms (GLI^A^), which then translocate to the nucleus and induce target genes expression. (Hh; hedgehog, PTCH1; Patched 1, SMO; Smoothened, GLI; gliomaassociated oncogene, GSK3β; glycogen synthase kinase 3β; PKA; protein kinase A, CK1; casein kinase 1, SUFU; Supressor of Fused, PKC; protein kinase C).

Hedgehog (Hh) signaling plays a key role during embryonic development and tissue patterning. The canonical pathway of the Hh signaling is initiated by the release of Hh ligands, namely Sonic Hh (SHH), Desert Hh (DHH), and Indian HH (IHH) [1]. In the absence of Hh ligands, the Hh receptor, Patched homolog 1 (PTCH1), prevents activation of the Hh pathway by suppressing the activity of the co-receptor Smoothened (SMO) [2]. Binding of the Hh ligand to the receptor leads to the accumulation of SMO and translocation of glioma-associated oncogene (GLI) transcription factors in a microtubule-based protrusion of the cell membrane–primary cilium [2,3,4]. GLI proteins belong to zinc finger transcription factors and are the main effectors of the Hh signaling. Three members of GLI transcription factors family (1–3) have been identified in vertebrates. In the primary cilium, GLIs dissociate from the negative regulator Suppressor of Fused (SUFU), are converted into their activator forms (GLI^A^) and translocate to the nucleus (Figure 1). Nuclear translocation of the GLI^A^ (GLI2^A^ and GLI3^A^) leads then to the expression of downstream targets, such as GLI1, cyclin D1, homeobox protein NANOG (NANOG), the inhibitory receptor PTCH1, and the decoy receptor hedgehog-interacting protein (HHIP) [5]. In the absence of ligand, SUFU directly binds GLI proteins and retains them in the cytoplasm, thus facilitating their processing into a repressor form (GLI^R^). Both GLI2 and GLI3 are subject to a limited proteolysis, giving rise to truncated repressor forms (GLI2^R^ and GLI3^R^). However, in comparison with GLI3, the proteolytic processing of GLI2 is much less efficient, with the majority of GLI2 being degraded. The repressor form translocates to the nucleus, where it competes with the activator form for the DNA-binding sites, thus hampering GLI target gene expression [6,7]. Posttranslational modifications, including phosphorylation by protein kinase A and C (PKA, PKC), casein kinase 1 (CK1), glycogen synthase kinase 3β (GSK3β), and dual-specificity Yak1-related kinase (DYRK1), have been shown to determine the activator versus repressor form of GLIs [8,9,10,11,12,13,14,15]. In addition to the canonical Hh signaling, a non-canonical, SMO-independent GLI activation has been recently described and will be discussed later in this review. 

Although most of the studies focused on the role of Hh signaling in the morphogenesis, this pathway is multifaceted and regulates a broad spectrum of other processes including tissue maturation, cell fate decisions (proliferation, apoptosis, migration, and differentiation), and maintenance of stem cell population [16,17,18,19,20]. In line with this notion, activation of the Hh signaling is not only a typical feature of embryogenesis, but it has been also observed in the postnatal period, where it maintains tissue homeostasis and drives repair and regeneration following injury [21,22,23]. Not surprisingly, the deregulation of Hh signaling may cause numerous disorders including birth defects, such as Gorlin syndrome and Greig cephalopolysyndactyly syndrome, as well as cancer [24,25,26,27,28,29,30]. Aberrant activation of the Hh pathway accounts for more than 25% of human cancer deaths [31]. Different types of cancer including pancreatic, basal cell carcinoma (BCC), medulloblastomas, gliomas, colorectal, prostate, lung, and breast cancer display abnormal Hh pathway activity [26,27,28,29,30,32,33,34]. Overactivation of the Hh pathway seen in cancer is a consequence of the following events: (i) excessive production of an Hh ligand resulting in an enhanced auto- as well as paracrine signaling; (ii) somatic mutations in the upstream pathway elements such as SMO and PTCH1; (iii) overexpression of the Hh pathway components (PTCH1, SMO, and GLI1); and (iv) the presence of an alternative splice variant of GLI1–termed truncated GLI1 (tGLI1).

The first hint, that the Hh pathway makes a critical contribution to cancer development, came from the studies describing mutations in PTCH1 gene in BCC [24]. This observation was reinforced by the discovery of other mutations in PTCH1, SMO, and SUFU in a large percentage of spontaneous BCCs and medulloblastomas [35,36]. Accordingly, transgenic mice with loss of PTCH1 or gain of function of SMO develop BCCs and medulloblastomas [37,38]. These genetic alterations lead to the hyperactivation of the Hh pathway and subsequent malignant transformation. 

However, mutations in PTCH1 and SMO are not the only reason why the Hh signaling is abnormally activated in tumors. Several human cancers display aberrant pathway activity in response to increased levels of a Hh ligand. These include multiple myeloma, gastric, breast, prostate, pancreatic, and bladder cancer [39,40,41,42,43,44]. Interestingly, Hh ligand produced by tumor cells may display effects on both tumor cells through autocrine signaling and tumor stroma (ligand responding cells) through paracrine signaling. An essential role of the Hh autocrine signaling has been shown in multiple myeloma, in which Hh ligand secreted by human myeloma cells enhanced their proliferation and protected them from spontaneous, as well as chemotherapy-induced, apoptosis in vitro and in vivo [42]. Similar mechanism has been reported for gastric cancer, in which the Hh autocrine signaling promoted tumor cell proliferation [45]. Furthermore, activation of the Hh pathway in lung cancer cells by an autocrine mechanism induced epithelial to mesenchymal transition, cell migration, and metastasis formation [46]. The paracrine type of the Hh pathway activation in tumor stroma cells also increased invasion and metastatic potential of tumor cells. An example of this is pancreatic cancer, in which Hh ligand produced by tumor cells activated the Hh signaling in stromal cells, which in turn supported the proliferation and invasion of the tumor, thus creating a positive feedback loop [47]. Paracrine Hh signaling has been further described in prostate, pancreatic, and lung cancer [47,48,49]. 

Overexpression of the Hh pathway components is the second most frequent reason for the pathway overactivation. Upregulation of SMO and Hh coreceptors [growth arrest-specific protein 1 (GAS1), brother of CDO (BOC), and cell adhesion molecule-related/down-regulated by oncogenes (CDON)] has been, for example, observed in cancer-associated fibroblasts derived from pancreatic adenocarcinomas [50,51]. Furthermore, a distinct subgroup of breast cancer (triple negative breast cancer/TNBC) characterized by high recurrence, metastasis formation, and, thus, a very poor prognosis, displayed aberrant Hh pathway activity caused by elevated expression of SMO and GLI1 [52]. Abnormal expression of the Hh pathway components has been further observed in lung, ovarian, gastric, and skin cancer [53,54,55]. A study by Onishi and colleagues tried to explain why the components of the Hh pathway exhibit increased expression in cancer cells. In this study, hypoxia induced invasiveness of different pancreatic ductal adenocarcinoma cells. Interestingly, this phenomenon strictly depended on Hh signaling, as inhibition of SMO abolished the hypoxia-triggered motility of the cells. The mechanism underlying this effect relied on the hypoxia-induced transcription of SHH, SMO, and GLI1, and thus a ligand-independent Hh pathway activation [56]. As hypoxia is a common characteristic of the growing tumors, the above mentioned mechanism may account for the excessive production of Hh pathway components in a wide variety of tumors.

Lastly, novel isoform of GLI1, termed truncated GLI (tGLI1), was discovered [57]. tGLI1 is an alternatively spliced, shorter variant of GLI1, and its expression is induced in glioblastomas and breast cancer. In comparison to GLI1, tGLI1 may induce the expression of several additional genes, including a cluster of differentiation 24 (CD24), histidinol phosphate aminotransferase 1 (HPA1), vascular endothelial growth factor C (VEGF-C), and tumor endothelial marker 7 (TEM-7). tGLI1 promotes proliferation, migration, and angiogenesis, and its overexpression correlates with larger tumors [57]. 

Although several mechanisms of Hh pathway activation seem to contribute to cancer development and progression, they all meet at the level of trancription factors–GLIs, which then execute a transcriptional response to the Hh signaling. The mechanism behind how GLIs may contribute to tumorigenesis involves the induction of the expression of genes belonging to a wide variety of pathways. For instance, GLI may induce expression of genes involved in (i) proliferation: Cyclin D1, Cyclin D2, N-MYC, insulin-like growth factor 2 (IGF2), and hairy and enhancer of split-1 (HES1); (ii) cell survival: B-cell lymphoma 2 (BCL2); (iii) angiogenesis: VEGF; (iv) genetic instability: p53 (v) epithelial-to-mesenchymal transition: zinc finger protein SNAI1 (SNAIL); and (vi) stem cell self-renewal: NANOG, sex determining region Y-box 2 (SOX2) [16,58,59,60,61,62]. Thus, a broad range of cancer-promoting processes seems to be regulated by GLIs. The involvement of the Hh pathway in the cancer development/progression gets even more complexed, considering the fact that this pathway may be activated by a non-canonical mechanism.

## 2. Mechanism of Hh Signaling Crosstalk with Other Pro-Tumorigenic Pathways

**Figure 2:**
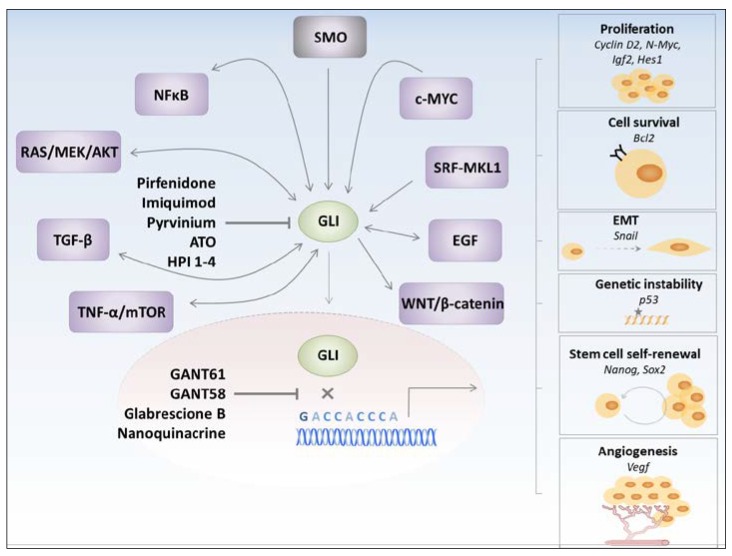
Crosstalk of the Hedgehog pathway with other protumorigenic pathways. GLI proteins may be activated through canonical (SMO-dependent) or non-canonical (SMO-independent) mechanisms. Multiple cancer-related signaling pathways may converge on and regulate GLIs, which represent molecular hubs governing various pro-tumorigenic processes such as proliferation, cell survival, epithelial-to-mesenchymal transition (EMT), genetic instability, stem cell self-renewal, and angiogenesis. Different possibilities to block GLIs are illustrated.

Interplay between various prooncogenic pathways is one of the reasons why target-specific chemotherapies display low efficacy or even relapse. Recent evidence suggests that the Hh pathway is one of the cascades actively involved in the net of perplexing interactions seen in cancer. Given the fact that the vast majority of cancers display aberrant Hh pathway activation [26,27,28,29,30], understanding the molecular mechanism underlying crosstalk between Hh signaling and other pathways is of utmost importance in order to design effective anti-cancer therapies. One of the underlying mechanisms leading to the crosstalk is a non-canonical mode of Hh pathway activation, which is Hh ligand- and SMO-independent [63,64,65,66,67,68,69]. Specifically, expression and activity of GLIs has been shown to be regulated by other prooncogenic pathways, thereby leading to the persistent activity of the Hh pathway. In addition to this mechanism, GLIs per se may induce gene expression or activate components of other pro-tumorigenic pathways, thus contributing to the complicated map of a tumor cell interactome (Figure 2). 

### 2.1. Non-Canonical Activation of the Hh Pathway

Growing body of evidence suggests that the potentiated expression and activity of GLIs is not only a result of the canonical but also non-canonical activation of Hh signaling. Strikingly, this becomes even more evident in the context of cancer development. For example, active crosstalk between TNF-α/mTOR/S6K1 and the Hh pathway has been observed in esophageal carcinoma. In this study, authors demonstrated that TNF-α potentiated GLI1 activity through S6K1-mediated phosphorylation of GLI1 at Ser84 in a SMO-independent manner. The crosstalk between TNF-α and the Hh pathway has been reinforced by the finding that blockage of the mTOR pathway enhances the suppressive effect of the Hh inhibitor in esophageal carcinoma [63]. Similar mechanism has been observed in pancreatic and gastrointestinal cancer cells [62,70]. The study by Singh and colleaques highlighted a critical role of DYRK1B kinase in the Hh/mTOR pathway crosstalk. Interestingly, this kinase displayed positive, as well as negative, effects on the Hh signaling. On one hand, DYRK1B blocked the signal transduction from SMO to GLIs, thus inhibiting the canonical Hh pathway activation; on the other hand, DYRK1B stimulated the mTOR/AKT pathway, which in turn promoted GLI stabilization and thus the non-canonical Hh signaling [68]. The NFκB pathway is another example of the cascade favoring GLIs activity. A study by Wang et al. showed that pro-inflammatory cytokines, such as TNF-α and IL-1β, can upregulate expression of GLI1 gene via NFκB and thus increase its abundance in the nucleus of pancreatic cancer cells [71]. Accordingly, a direct binding of the NFκB transcriptional complex to the GLI1 promotor region has been observed, and inhibition of NFκB decreased GLI1 expression in breast cancer cells [72]. 

Other pro-tumorigenic pathways, which regulate GLIs expression and activity, are RAS/MEK/AKT, the c-MYC, TGF-β, and the serum response factor (SRF)/megakaryoblastic leukemia 1 (MKL1) pathway. RAS, MEK, and AKT can increase the nuclear presence of GLI1 [73]. RAS may also induce expression and activity of GLIs by utilizing RAF/MEK/MAPK axis [29]. An interesting observation has been made by Lauth and colleagues, who discovered a RAS-dependent shift from autocrine to paracrine Hh activation. In this study, RAS induced expression of the Hh ligand, which in turn potentiated Hh pathway activation in tumor stroma but at the same time blocked the Hh signaling in tumor cells. These effects have been shown to be regulated by RAS effector molecule-DYRK1B [74]. c-MYC, one of the most frequently deregulated transcription factors in cancer, has been found to regulate GLI1 expression, thus supporting an antiapoptotic role of GLI1 in Burkitt lymphoma [75]. Finally, a number of studies, including ours, demonstrated crosstalk between TGF-β signaling and GLI expression and activity. TGF-β has been shown to induce expression of GLI1 and GLI2 [64,65], the latter mediated by the recruitment of SMAD3 and β-catenin to the distinct elements of the GLI2 promotor. Interestingly, TGF-β-induced expression of GLI1 was strictly GLI2 dependent and insensitive to cyclopamine (a SMO inhibitor) treatment, further demonstrating a non-canonical method of the activation of the Hh pathway. Consistently, GLI1 and GLI2 levels were inhibited by an activin receptor-like kinase (ALK5; TGF-β type I receptor) small molecule inhibitor [65,66]. Another study demonstrated that bone morphogenetic protein type I receptors a (Bmpr1a) and b (Bmpr1b) mutant mice developed granulosa cell tumors, which exhibited aberrant TGF-β signaling associated with increased expression of GLI1 and GLI2 [76]. Furthermore, TGF-β, by the inhibition of PKA, may also affect the stability of GLI proteins and in consequence lead to their accumulation in tumor cells [67], enhancing their growth, proliferation, and invasiveness [65,66]. Finally, SRF-MKL1 can amplify GLI1 transcriptional activity by forming a complex with this protein. This may result in the increased viability of tumor cells [77]. Altogether, these results support the idea that GLI proteins rather than the Hh pathway per se play an important role in tumorigenesis.

### 2.2. GLIs-Dependent Regulation of Pro-Tumorigenic Pathways

Employing GLI proteins by other pro-tumorigenic pathways is one of the mechanisms underlying the crosstalk with the Hh pathway. In addition to this mechanism, GLI proteins may also regulate other pro-tumorigenic pathways by inducing expression of genes or by interacting with the components of the related pathways (Figure 2). Interestingly, in most of the cases, communication between the two pathways leads to a positive feedback loop, in which activation or accumulation of a component from the first pathway leads to the activation or accumulation of another component belonging to a second pathway. This creates a vicious cycle, which enhances cancer development, progression, and metastasis. An example of such a vicious cycle has been described for the TGF-β and the Hh pathway. In the study by Fan et al., tumor cells, which exhibited high levels of active SMO, also displayed high TGF-β2 expression and activity of the TGF-β-ALK5-Smad3 signaling. Conversely, inhibition of TGF-β receptor I reduced tumor area in a mouse model of SMO-induced BCC [78]. In gastric cancer, SHH-triggered tumor cell invasion was associated with the activation of the TGF-β signaling pathway, and the blockage of the TGF-β signaling inhibited SHH-induced cell motility [66]. Thus, the mutual crosstalk between the Hh and TGF-β pathways may efficiently accelerate cancer progression.

An example of a positive feedback loop has been also observed between the Hh and the AKT, as well as the Hh and the NFκB pathways. As mentioned above, AKT may modulate GLI activity, and GLI can control AKT expression by binding to the AKT promotor region [79]. Similarly, NFκB seems to regulate GLI expression via direct interaction with GLI promotor region, and GLI utilizes the I-kappa-B kinase epsilon (IKBKE) to increase the NFκB pathway activity and, in consequence, accelerate pancreatic tumor formation [80]. 

A special type of communication has been described between GLI proteins and an epidermal growth factor (EGF) signaling. Namely, simultaneous activation of the Hh/GLI and the EGF pathway synergistically induced oncogenic transformation of human keratinocytes, and this effect was dependent on the activation of RAS/RAF/MEK/ERK axis. Correspondingly, combined treatment of cancer cells with a GLI antagonist (GANT61) and an EGF inhibitor (gefitinib) reduced tumor cell proliferation more efficiently than the treatment with each inhibitor alone [81]. Another group demonstrated that EGF and Hh signaling may merge at the level of promotors and synergistically induce expression of selected genes such as interleukin-1 receptor type 2 (IL1R2), Jagged 2 (JAG2), cyclin D1, S100 calcium-binding protein A7 (S100A7), and A9 (S100A9). Pharmacological inhibition of EGFR and ERK1/2 abrogated expression of GLI/EGF target genes, suggesting that EGF can signal via MEK/ERK to cooperate with GLI proteins in regulation of target gene expression [82].

A relationship between WNT/β-catenin signaling and GLIs in cancer has been demonstrated as well, and it seems to involve complexed interactions. It has been shown that GLI1 potentiates β-catenin activity indirectly by inducing the expression of SNAIL, Proto-Oncogene Int-1 Homolog (WNT), secreted frizzled related protein 1 (sFRP1), and mucin-5 subtype AC [83,84,85] or directly by promoting the synthesis of WNT ligands 2B, 4, and 7B [86]. Other studies, however, demonstrated the opposite effects. For example, it has been shown that GLI1 uses FRP1 [87,88] to inhibit the activity of β-catenin [89] and that a Hh ligand reduces the expression of WNT5A by increasing the synthesis of forkhead transcription factors in embryonic intestine [90]. Thus, reciprocal interactions between the Hh and WNT/β-catenin pathways seem to be more complexed and content-dependent. 

The above-mentioned examples demonstrate that multiple cancer-related signaling pathways converge on and regulate GLIs (Figure 2). Moreover, GLIs may respond back and generate positive feedback loops in order to promote and amplify pro-tumorigenic insults. Thus, GLI transcription factors represent molecular hubs governing numerous cancer-related signaling arms. This provides a strong rationale to target GLIs, rather than other Hh pathway components, in order to inhibit canonical and non-canonical inputs operating through GLIs, as well as GLI-dependent tumor-promoting outputs. 

## 3. GLI Transcription Factors Inhibitors

A number of inhibitors targeting the Hh signaling pathway are nowadays available at the market (Table 1) [91]. Most of these inhibitors belong to a group of SMO inhibitors, and two of them (vismodegib and sonidegib) reached FDA aproval for the treatment of locally advanced and metastatic BCC [92,93]. Although both of the inhibitors display very good response rates in BCC patiens leading to tumor shrinkage and reduced progression [92,94,95,96], many patients experience recurrence after discontinuation of the treatment [97,98]. Acquired mutations of SMO [99,100] and the fact that the Hh signaling can be activated downstream of SMO may explain this adverse effect. This notion is further supported by the findings that demonstrated that BCCs resistant to SMO inhibitors respond to GLI blockers. This provides a rationale for using GLI inhibitors in vismodegib-resistant BCC patients [100].

GANT61 and GANT58 are small molecule GLI antagonists, which interfere with the binding of GLIs to DNA, though GANT61 displays higher specificity to GLIs and more efficiently blocks GLIs binding to DNA [101]. These inhibitors block tumor cell proliferation in vitro and suppress tumor cell growth in vivo. For example, GANT61 demonstrated a GLI-specific antitumor activity in lung carcinoma xenograft model [102], acute myeloid leukemia [103], rhabdomyosarcoma [104], neuroblastoma [105], breast cancer [106], and pancreatic cancer [101]. Notably, these studies also showed substantial benefit of GANT61 over SMO inhibitors, further confirming critical role of GLIs in cancer [101,102,106]. A similar mode of action has been described for glabrescione B, a small molecule GLI1 inhibitor, which binds at the interface of zinc finger domain 4 and 5 of GLI1. As a consequence, glabrescione B prevents binding of GLI1 to DNA, impairs its transcriptional activity, and reduces the growth of Hh-dependent tumor cells in vitro and in vivo, as well as the self-renewal ability and clonogenicity of tumor-derived stem cells [107].

Arsenic trioxide (ATO) is an FDA-approved drug used for the treatment of acute promyelocytic leukemia, as a second line of therapy for patients who do not respond or relapse to trans-retinoic acid therapy [108]. This drug displays a broad spectrum of anti-tumor activities, and its mechanism of action seems to involve several targets, such as promyelocytic leukemia–retinoic acid receptor α fusion protein, NF-κB, thioredoxin reductase, and JNK [109,110,111,112,113,114]. In addition, this drug directly interacts with GLIs and consequently inhibits GLI target gene expression. In detail, ATO reduces stability of GLI2 and thus growth of the Hh pathway-driven medulloblastoma allografts [115]. Given the fact that GLI2 connects various pro-tumorigenic pathways, one may speculate that the beneficial effect of ATO can be also attributed to its ability to block GLI2 and thus other oncogenic insults. Our findings demonstrate a similar mechanism of action for pirfenidone. Pirfenidone is an anti-fibrotic drug approved for the treatment of idiopathic pulmonary fibrosis (IPF) [116]. This drug displays broad spectrum of activities, including anti-inflammatory, anti-oxidant, and anti-fibrotic effects [117]. Importantly, we could demonstrate that pirfenidone selectively destabilizes GLI2 and thus blocks the Hh and the TGF-β-driven effects [64]. As both of these pathways favor tumorigenesis, the anti-tumor effects of pirfenidone on Hh/GLI-driven cancers are expected. Indeed, few recent studies provided evidence that pirfenidone is a promissing anti-tumor agent for lung, breast, hepatocellular, and pancreatic cancer, and malignant gliomas [118,119,120,121,122,123,124]. In addition, a study by Miura et al. demonstrated reduced incidence of lung cancer in IPF patients who underwent pirfenidone treatment [120]. 

Additional inhibitors that target GLI include Hh pathway inhibitors (HPI1, HPI2, HPI3, and HPI4), pyrvinium, imiquimod, and nanoquinacrine. HPIs have been shown to act downstream of SMO, influencing stability, degradation rate of GLIs, and trafficking of GLIs to the primary cilium [125]. Pyrvinium is an anti-helmetic drug, which has been demonstrated to reduce the stability of the GLI transcription factors. By acting as an allosteric activator of CK1 [126], pyrvinium facilitates the association of CK1 with GLI1, its subsequent phosphorylation, and, finally, proteasomal degradation [127]. Another example of a drug modulating GLI stability is imiquimod. Imiquimod is an agonist of the toll-like receptor (TLR) 7 and the TLR8, which, via the interaction with adenosine receptors (ADORAs), activates PKA, thus favoring GLI2 phosphorylation and subsequent degradation [128]. Lastly, nanoquinacrine (NQC), a spherical nanoparticle form of quinacrine (QC; an anti-cancer drug), has been shown to interfere with the Hh/GLI pathway in multiple ways. NQC increases expressions of negative regulators of GLI, GSK3β, and PTEN and, in addition, destabilizes a GLI1-DNA complex by interfering with the binding of GLI1 to its consensus DNA binding sequence (5′GACCACCCA3′). Consequently, it has been demonstrated that NQC reduces GLI1-dependent proliferation and tumor growth [129]. 

From the above-listed GLI inhibitors, only ATO, pirfenidone, and imiquimod reached clinical trials in different types of cancer. Besides leukemia, ATO has been also tested in BCC patients who experienced relapse after an SMO inhibitor therapy. This study demonstrated reduction of GLI1 mRNA levels in the patient’s biopsies. Although some patients experienced stable disease for the period of 3 months, none had tumor shrinkage. This could have be explained by the sequential dosing of ATO (5 doses every 28 days) and thus an incomplete Hh pathway inhibition [130]. Therefore, future studies using continuous dosing of ATO to achieve a full inhibition of the Hh pathway are required to decipher the possible benefit from ATO administration in patients suffering from BCC. In the case of pirfenidone, phase II clinical trial showed a benefit from pirfenidone intake in patients with neuroblastoma. Most of the patients experienced longer progression-free period, and some patients had a decrease in tumor volume by 15% [131]. An ongoing study aiming at testing pirfenidone efficacy in patients suffering from different types of lung cancer is currently recruiting the subjects (www.clinicaltrials.gov, ID: NCT03177291). Imiquimod has been tested in BCC patients, as well as in breast and cervical cancer [132,133]. In breast cancer, topical application of imiquimod was well tolerated and induced the disease regression; however, in cervical cancer the results were contradictory. In some cases, imiquimod had no effect or even worsened the disease [134]; in other cases, imiquimod efficiently promoted cancer regression [135]. Future studies have to clarify whether these effects are related to imiquimod’s immunomodulatory functions, or rather its ability to interfere with the Hh signaling.

Although several lines of evidence suggest beneficial effects resulting from GLI inhibition, one should consider that this pathway actively participates in the regulation of many processes that are required for proper tissue regeneration and repair. Thus, inhibition of Hh/GLI signaling may eventually lead to disease progression, as has been already demonstrated for diverse organ injuries [21,136,137,138,139,140,141]. In line with this notion, few studies aiming at GLI inhibition in pancreatic, bladder, and colon cancer supported an anti-oncogenic rather than a cancer-promoting function of the Hh/GLI pathway. These anti-oncogenic effects could be partially explained by a complex interplay between tumor cells and stroma. For instance, activation of the Hh pathway induced BMP signaling in the bladder cancer stroma, which in turn effectively blocked tumor growth [142]. Conversely, blockage of the Hh signaling in the stroma accelerated progression of the bladder carcinoma. A similar tumor-restraining effect of the Hh/GLI signaling has been observed in pancreatic cancer, in which the increase in the number of GLI1-expressing stromal cells positively correlated with the reduced growth of the tumor [143,144]. Furthermore, stroma-specific activation of the Hh/GLI pathway reduced the tumor load of colon cancer via the activation of the BMP signaling and the reduction of tumor-derived stem cells [145]. Lastly, the Hh/GLI protective role has been described in a colitis-induced adenocarcinoma. Activation of the Hh/GLI pathway in the tumor stroma attenuated colitis and limited the colitis-induced adenocarcinoma development by inducing the expression of IL-10, an immune-modulatory cytokine known to suppress inflammatory intestinal damage [146]. Hence, understanding the interactions between the tumor cells and the stroma, which may elicit tumor-promoting and restrictive effects, is crucial for the development of effective anti-cancer therapies. 

## 4. Hh Pathway in Cancer Stem Cells

Metastasis and relapse after successful eradication of the primary tumor are two major challenges for a cancer therapy. Until now, it remains to be seen why after a long tumor-free period, some patients experience cancer recurrence. One of the hypotheses suggests a presence of a special subset of cancer cells, which possess characteristics of stem cells. Cancer stem cells (CSC) have the ability to give rise to all cell types found in the bulk of the tumor, and thus are able to initiate the formation of the secondary tumor [160]. Numerous studies indicate that the Hh pathway drives CSC maintenance in lung, breast, prostate, pancreas, and colon cancers, as well as glioblastoma, multiple myeloma, and chronic myelogenous leukemia [39,161,162,163,164,165,166,167]. GLI proteins contribute to the maintenance of CSC population by regulating the expression of CSC stemness genes, such as NANOG, octamer binding transcription factor 4 (OCT4), SOX2, WNT-2, CD44, and Kruppel-like factor 4 (KLF4) [62,168]. Accordingly, the inhibition of the Hh pathway in medulloblastoma supressed proliferation of CSC and its clonogenic self-renewal ability [169]. Similarly, Singh et al. demonstrated that the Hh pathway inhibition increases apoptosis of pancreatic CSC [170]. In line with these results, siRNA directed against GLI1 and GLI2 diminished polycomb complex protein (BMI)-1-mediated self-renewal capacity of CSC [164]. Selective activation of the Hh pathway in CSC as compared to the bulk of tumor cells [39,171,172,173,174,175,176] was further supported by Varnat et al., who analyzed colon carcinomas from 40 patients and confirmed a consistent increase of GLI1 levels in CSC over the course of metastasis formation [177]. Interestingly, the same group also demonstrated that CSC undergo a molecular switch following metastatic transition and are characterized by the suppressed activity of the WNT signaling pathway and enhanced activity of the Hh signaling [168].

## 5. Future Directions

To summarize, numerous lines of evidence support the blockage of GLI transcription factors as a therapeutic strategy for cancer. Firstly, though SMO inhibitors have been successfully used as monotherapy in BCC, recurrence of the disease after discontinuation of the treatment due to acquired resistance is high. At this point, GLI inhibitors could be used as a second line therapy to repress active Hh pathway in SMO-resistant cancer cells. Secondly, crosstalk between Hh and other signaling pathways at the level of GLI limits the use of SMO inhibitors. Suppression of GLIs offers a possibility with which to combat different upstream oncogenic insults, and thus block canonical, as well as non-canonical, inputs. Thirdly, GLI inhibitors would not only be beneficial to treat primary but also secondary tumors, as they regulate CSC maintenance. Taken together, a large number of studies demonstrated the superior role of GLI transcription factors in cancer development, progression, and metastasis formation, thus making them an attractive therapeutic target for anti-cancer therapy, worthy of being tested in future clinical trials.

## Figures and Tables

**Table 1:** List of Hh pathway inhibitors.

Inhibitor Name	Target	Mode of Action	Reference
Robotnikinin	SHH	binds to the N terminal part of SHH	[147]
RU-SKI 43	SHH	inhibits SHH palmitoylation	[148]
5E1 antibody	SHH	binds to the N terminal part of SHH	[149]
GDC-0449 (Vismodegib)	SMO	antagonist of SMO	[150]
LDE225 (Sonidegib)	SMO	antagonist of SMO	[151]
IPI 926 (Saridegib)	SMO	cyclopamine-derived antagonist of SMO	[152]
LY2940680 (Taladegib)	SMO	antagonist of SMO	[153]
PF-04449913 (Glasdegib)	SMO	antagonist of SMO	[154]
Cyclopamine	SMO	antagonist of SMO, blocks conformational change of SMO into the active form	[155]
TAK-441	SMO	antagonist of SMO	[156]
CUR61414	SMO	antagonist of SMO	[157]
Jervine	SMO	antagonist of SMO, blocks conformational change of SMO into the active form	[158]
BMS-833923	SMO	antagonist of SMO	[159]
GANT61, GANT58	GLI	blocks binding of GLIs to DNA	[101]
Glabrescione B	GLI	blocks binding of GLIs to DNA	[107]
Arsenic trioxide	GLI	reduces stability of GLI2	[115]
Pirfenidone	GLI	reduces stability of GLI2	[64]
HPI 1–4	GLI	influence stability, degradation rate, and trafficking of GLIs to the primary cilium	[125]
Pyrvinium	GLI	induces proteosomal degradation of GLIs through CK1–mediated phosphorylation	[127]
Imiquimod	GLI	induces proteosomal degradation of GLIs through ADORA/PKA–mediated phosphorylation	[128]
Nanoquinacrine	GLI	increased expressions of GSK3β, PTEN and binds to and destabilizes GLI1-DNA complex	[129]
